# Clinical and Microbiological Characteristics of Septic Arthritis in 25 Adults at a Tertiary Hospital in Cambodia: A Single-Center Retrospective Case Series

**DOI:** 10.7759/cureus.116082

**Published:** 2026-09-10

**Authors:** Theara Phina, Pheng Ann Kao, Vireak Khun, Veasna Heng, Ponndara Ith, Setha Limsreng

**Affiliations:** 1 Outpatient Department, Luang Mè Hospital, Phnom Penh, KHM; 2 Medicine A Department, Calmette Hospital/University of Health Sciences, Phnom Penh, KHM; 3 Department of Medicine, Preah Ang Duong Hospital, Phnom Penh, KHM; 4 Traumato-Orthopedic Unit, Calmette Hospital, Phnom Penh, KHM; 5 Faculty of Medicine, University of Health Sciences, Phnom Penh, KHM; 6 Department of Medicine B-A5 Ward, Calmette Hospital/University of Health Sciences, Phnom Penh, KHM

**Keywords:** burkholderia pseudomallei, cambodia, melioidosis, retrospective study, septic arthritis

## Abstract

Introduction

Septic arthritis (SA) is a potentially destructive joint infection requiring timely diagnosis and treatment. Published adult data from Cambodia are limited, and local microbiological patterns may differ from those reported in other settings.

Objective

The objective of this study is to describe the demographic, clinical, laboratory, and available microbiological characteristics of adults treated for SA in a tertiary hospital in Cambodia.

Materials and methods

We conducted a small, single-center retrospective descriptive case series of 25 adults admitted to Medicine Ward B-A5, Calmette Hospital, from January 1, 2023, through December 31, 2024. Data were abstracted from medical records. Microbiological results were recorded as documented in the charts; detailed specimen-processing, organism-identification, and antimicrobial-susceptibility methods were not consistently available. Prior antibiotic exposure before presentation and treatment outcomes were also not consistently documented and were not analyzed.

Results

Among 25 patients, 15 (60%) were male, and the mean age was 49.52 ± 12.24 years. Ten (40%) were farmers and 21 (84%) lived outside Phnom Penh. Diabetes mellitus and alcohol use were each documented in 12 patients (48%). Joint pain (20/25, 80%), fever (16/25, 64%), and joint swelling (15/25, 60%) were common. The knee was affected in 17 patients (68%). An organism was reported from blood and/or synovial fluid culture in 13 patients (52%). *Burkholderia pseudomallei* was reported in five patients, methicillin-susceptible *Staphylococcus aureus* in three, *Mycobacterium tuberculosis* in two, and *Escherichia coli*, methicillin-resistant *S. aureus*, and *Acinetobacter baumannii* in one patient each. These organism frequencies should be interpreted as record-based observations rather than estimates of pathogen distribution.

Conclusion

This small single-center series describes the clinical experience of 25 adults with SA at a Cambodian tertiary referral hospital. The frequent recording of *B. pseudomallei* is clinically notable in the context of recognized melioidosis in Cambodia, but the small sample, referral setting, culture-negative cases, and incomplete microbiological documentation preclude conclusions about national pathogen prevalence. Larger multicenter studies using standardized microbiological methods and systematic treatment and outcome data are needed.

## Introduction

Septic arthritis (SA) is an infection of a joint caused by bacteria, viruses, or fungi, commonly caused by bacterial invasion, and can lead to potential joint destruction if untreated. It is a medical emergency requiring prompt diagnosis and treatment. SA typically presents as monoarticular but can also manifest as polyarticular. The knee is more commonly affected compared to other joints. An infection of a native joint can be classified into acute (< three weeks) and chronic (> three weeks) categories [1]. The incidence of proven and probable SA in Western Europe is 4-10 cases per 100,000 people [2], and it seems to be rising. Predisposing factors for the development of SA include rheumatoid arthritis or osteoarthritis, joint prosthesis, low socioeconomic status, alcoholism, diabetes, previous intra-articular corticosteroid injection, cutaneous ulcers, and immunosuppressive treatment [2]. Diagnosis of SA can be challenging even for doctors skilled in the management of musculoskeletal disease. The most common symptoms are fever, joint pain, swelling, warmth, and reduced joint mobility [1,2]. Most cases of SA are caused by bacteria including *Staphylococcus aureus*, *Streptococcus pneumoniae*, *Burkholderia pseudomallei*, *Mycobacterium tuberculosis*, *Escherichia coli*, *Neisseria gonorrhoeae,* and other nongonococcal bacteria. However, in SA, *S. aureus* is the most common organism [3]. Joint aspiration and blood culture are systematically performed in any case of suspected SA. Results from synovial fluid and blood cultures are definitively pathogen. However, in nearly 20% of cases, synovial fluid cultures remain negative even when SA is suspected [4]. Management of SA involves antibiotic therapy or joint drainage. Antibiotic therapy must be selected based on the antibiogram. Empiric antibiotic therapy should be started while awaiting culture results and following local guidelines. The joint should be aspirated to dryness and surgical drainage/washout, or daily arthrocentesis may be required [5].

Despite the clinical importance of SA, data on its epidemiology, clinical characteristics, microbiological profile, and associated factors in Cambodia remain limited. In particular, there is a lack of published data describing the characteristics of patients with SA and the causative microorganisms identified in Cambodian hospitals. This limited local evidence may make it difficult to understand the clinical and microbiological patterns of SA in the Cambodian population and to guide appropriate diagnostic and therapeutic strategies. Therefore, we conducted this study to describe the demographic characteristics, clinical manifestations, laboratory findings, microbiological results, and predisposing factors among patients with SA admitted to our hospital. The findings may provide useful baseline information on SA in Cambodia and contribute to improving its recognition and management in clinical practice.

## Materials and methods

Study design and setting

This was a single-center retrospective descriptive case series based on medical records from Medicine Ward B-A5 at Calmette Hospital, a tertiary referral hospital in Phnom Penh, Cambodia. The study period was January 1, 2023, through December 31, 2024.

Case identification and eligibility

Hospital records identified 26 adults diagnosed with or suspected of having SA during the study period. Records were eligible when patients were aged 18 years or older and had either (1) a microorganism reported from synovial fluid culture, (2) a microorganism reported from blood culture in a clinically compatible case, or (3) no microbiological identification but clinical, synovial-fluid, laboratory, and/or imaging findings considered compatible with SA by the treating team. Records were excluded for incomplete documentation that prevented assessment, rheumatic fever, monoarticular rheumatoid arthritis, or another inflammatory arthritis judged to better explain the presentation. One record was excluded because of incomplete documentation, leaving 25 patients.

Case classification

For this retrospective review, culture-positive SA was defined as a clinically compatible case in which a pathogenic organism was reported from synovial fluid and/or blood culture. Culture-negative SA was defined as a clinically compatible case without an organism reported from these cultures. Because the study relied on existing records, the diagnostic classification reflects the treating team's documented assessment and the data available in the chart.

Data collection

A structured data collection form was used to abstract demographic characteristics, residence, occupation, predisposing conditions, clinical manifestations, affected joint, blood laboratory results, synovial-fluid findings, and recorded microbiological results. Data on antibiotic use before hospital presentation were not consistently available. In-hospital treatment, complications, discharge status, mortality, recurrence, and longer-term outcomes were also not consistently documented in the dataset and therefore were not analyzed.

Microbiological data

Synovial-fluid and blood-culture results were transcribed from the medical records. The charts did not consistently document specimen volume, timing relative to antibiotic administration, transport conditions, culture media, incubation duration and atmosphere, organism-identification procedures, confirmatory testing for *B. pseudomallei *or *M. tuberculosis*, or the standards and methods used for antimicrobial-susceptibility testing. Consequently, organism identifications are reported as documented in the clinical records, and the study cannot independently verify the laboratory methods. Detailed susceptibility percentages were not used for inference because testing methods and denominators were incompletely documented.

Statistical analysis

Data were entered into Microsoft Excel 2016 (Microsoft Corporation, Redmond, USA) and summarized descriptively. Categorical variables are presented as counts and percentages. Continuous variables are presented as means with standard deviations when available. No inferential statistical testing was planned because of the small descriptive sample.

Ethical statement

The study protocol was reviewed and approved by the National Ethics Committee for Health Research (NECHR), Ministry of Health, Cambodia (approval no. 382 NECHR). As this was a retrospective study based on existing medical records, patient confidentiality and privacy were maintained throughout data collection and analysis. Personal identifiers were not included in the analyzed dataset or reported in this manuscript.

## Results

Study population and demographic characteristics

Twenty-six records were assessed for eligibility; one was excluded because the medical record was incomplete, and 25 patients were included (Figure 1). Eleven patients (44%) were treated in 2023 and 14 (56%) in 2024. Fifteen patients (60%) were male and 10 (40%) were female. The mean age was 49.52 ± 12.24 years (range: 22-75 years). Ten patients (40%) were farmers. Twenty-one (84%) lived in provinces outside Phnom Penh and four (16%) lived in Phnom Penh (Table 1).

**Table 1:** Demographic characteristics of the 25 included patients.

Characteristic	Result
Number of patients	25
Year of diagnosis	2023: 11 (44%); 2024: 14 (56%)
Sex	Male: 15 (60%); Female: 10 (40%)
Male-to-female ratio	1.5:1
Mean age	49.52 ± 12.24 years
Age range	22-75 years
Largest occupational group	Farmers: 10 (40%)
Residence	Outside Phnom Penh: 21 (84%); Phnom Penh: 4 (16%)

**Figure 1:**
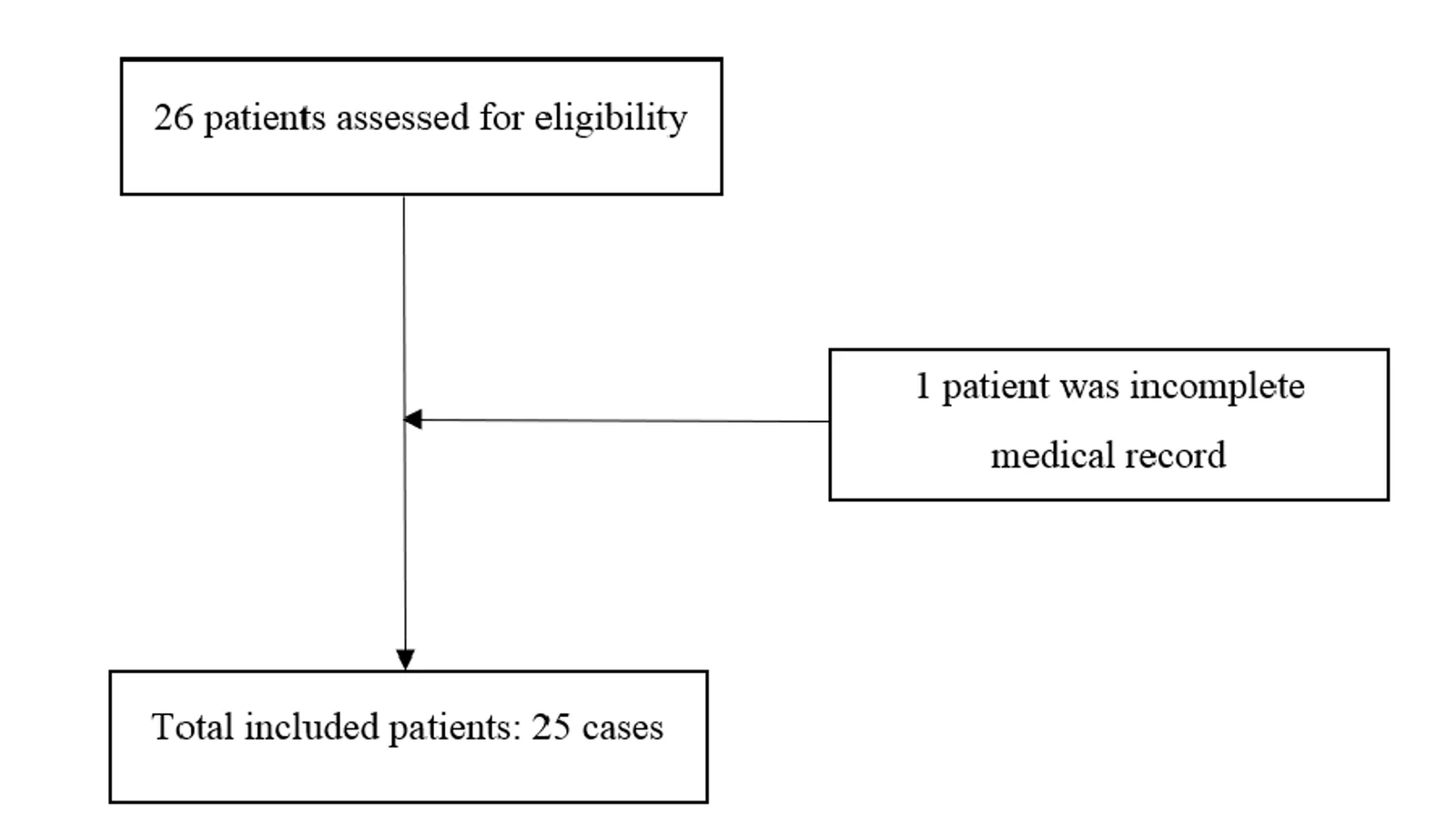
Study flow diagram. Twenty-six records were assessed for eligibility; one was excluded because of an incomplete medical record, leaving 25 patients in the retrospective case series.

Predisposing factors and clinical manifestations

Type 2 diabetes mellitus and alcohol use were each documented in 12 patients (48%). Pre-existing joint disease was recorded in six patients (24%), immunosuppressive therapy in four (16%), age >60 years in four (16%), chronic kidney disease in three (12%), and chronic liver disease in one patient (4%). Joint pain was documented in 20 patients (80%), fever in 16 (64%), joint swelling in 15 (60%), and joint dysfunction in two (8%). The knee was affected in 17 patients (68%), followed by the hip in three (12%), ankle in two (8%), elbow in two (8%), and shoulder in one (4%) (Table 2).

**Table 2:** Predisposing factors, clinical manifestations, and affected joints.

		n	%
Predisposing factor	Type 2 diabetes mellitus	12	48
	Alcohol use	12	48
	Pre-existing joint disease	6	24
	Immunosuppressive therapy	4	16
	Age >60 years	4	16
	Chronic kidney disease	3	12
	Chronic liver disease	1	4
Clinical manifestation	Joint pain	20	80
	Fever	16	64
	Joint swelling	15	60
	Joint dysfunction	2	8
Affected joint	Knee	17	68
	Hip	3	12
	Ankle	2	8
	Elbow	2	8
	Shoulder	1	4

Laboratory findings

The mean hemoglobin concentration was 11.48 ± 1.34 g/dL; 18 patients (72%) had a value below 12 g/dL and seven (28%) had a value at or above 12 g/dL. The mean peripheral WBC count was 10.42 ± 3.26 G/L (range 4.82-16.64 G/L); 13 patients (52%) had values >9 G/L and 12 (48%) had values <9 G/L. The mean CRP was 115.33 ± 61.03 mg/L. Synovial fluid was described as cloudy in 20 patients (80%), purulent in three (12%), and reddish in two (8%). All 25 patients had a recorded synovial-fluid WBC count >2,000 cells/mm³, and the reported mean was 38,500 cells/mm³ (Table 3). The contradictory CRP range/category values in the source draft were not reproduced because they require verification against the original dataset.

**Table 3:** Laboratory and synovial-fluid findings. CRP category/range values from the source draft were omitted pending source-data verification.

Domain	Measure	Result
Blood	Hemoglobin	Mean 11.48 ± 1.34 g/dL; <12 g/dL: 18 (72%); ≥12 g/dL: 7 (28%)
	WBC count	Mean 10.42 ± 3.26 G/L (range 4.82-16.64); >9 G/L: 13 (52%); <9 G/L: 12 (48%)
	CRP	Mean 115.33 ± 61.03 mg/L
Synovial fluid	Appearance	Cloudy: 20 (80%); purulent: 3 (12%); reddish: 2 (8%)
	WBC count	>2,000 cells/mm³: 25 (100%); reported mean 38,500 cells/mm³

Microbiological findings

An organism was reported from blood and/or synovial fluid cultures in 13 of 25 patients (52%); 12 (48%) had no organism reported. Blood cultures were reported positive in 11 patients (44%), synovial-fluid cultures in 10 (40%), and both in eight (32%) (Table 4). Across the 25-patient series, the organisms documented in the records were *B. pseudomallei* in five patients (20%), *methicillin-susceptible S. aureus* (MSSA) in three (12%), *M. tuberculosis* in two (8%), *E. coli* in one (4%), methicillin-resistant S. aureus (MRSA) in one (4%), and A. baumannii in one (4%). Because laboratory identification methods were incompletely documented, these should be considered recorded organism identifications rather than independently verified etiologic assignments.

**Table 4:** Recorded culture results.

Culture result	n	%
Organism reported from blood and/or synovial fluid	13	52
No organism reported	12	48
Blood culture positive	11	44
Synovial-fluid culture positive	10	40
Both blood and synovial-fluid cultures positive	8	32

Antimicrobial susceptibility data

Antimicrobial susceptibility results were available for some bacterial isolates in the reviewed medical records. However, detailed information regarding the susceptibility testing methods, testing platform, interpretive standards, and antibiotics tested for each isolate was not consistently documented. Therefore, these data were considered insufficient for reliable estimation of antimicrobial susceptibility or resistance patterns and were not analyzed further.

## Discussion

This small retrospective series describes 25 adults treated for SA in a single tertiary referral ward in Phnom Penh. The mean age was approximately 50 years, 60% were male, and the knee was the most frequently affected joint. These broad clinical patterns are compatible with prior adult series from India, Singapore, Türkiye, and New Zealand, although direct comparisons should be interpreted cautiously because study designs, case definitions, referral patterns, and microbiological practices differed [6-9].

Diabetes mellitus and alcohol use were each documented in nearly half of the patients. Diabetes is a recognized risk factor for invasive infection and is particularly relevant to melioidosis. However, because this study did not include a control group, these frequencies should not be interpreted as measures of association or causation.

The record-based microbiological findings deserve cautious attention. *B. pseudomallei* was documented in five of the 25 patients and was the most frequently recorded organism in this series. Cambodia is a recognized setting for melioidosis: a Phnom Penh series described 58 adults with culture-confirmed melioidosis and reported diabetes as a common risk factor, rainy-season clustering, and bone or joint involvement in a subset of patients [10]. Other Cambodian studies have documented pulmonary melioidosis in provincial hospitals, pediatric disease in northern Cambodia, and culture-confirmed cases in Takeo Province [11-13]. This local literature makes the presence of *B. pseudomallei* in a Cambodian SA series biologically and epidemiologically plausible.

Nevertheless, the present data cannot establish that *B. pseudomallei *is the leading cause of SA in Cambodia. Only 25 patients from one referral ward were included, almost half of the series was culture-negative, and the medical records did not consistently contain the laboratory details needed to independently assess organism identification. Referral bias is also likely: a tertiary hospital may receive more severe, unusual, or diagnostically complex infections than primary or provincial facilities. The five recorded *B. pseudomallei* cases should therefore be presented as a clinically notable signal that supports awareness and further study, not as a national pathogen-prevalence estimate.

The culture-negative proportion was 48%. Several factors can contribute to negative cultures, including prior antimicrobial exposure, specimen timing and volume, transport, culture conditions, and nonbacterial or fastidious organisms. In this dataset, antibiotic exposure before specimen collection was not consistently documented, so its contribution cannot be assessed. This missing information is important because pretreatment can reduce culture yield.

Treatment and outcome data were not consistently available in the study dataset. Accordingly, this series cannot evaluate treatment adequacy, surgical management, complications, length of stay, mortality, relapse, or functional outcome. These variables are especially important for SA and melioidosis and should be collected systematically in future work.

Limitations

The principal limitations are the small sample size, single-center retrospective design, use of records from one tertiary referral ward, and possible selection and referral bias. The case definition depended partly on clinical documentation for culture-negative cases. Detailed microbiological methods were not consistently available, and nearly half of patients had no organism reported. Prior antibiotic exposure, timing of cultures relative to treatment, detailed antimicrobial-susceptibility methods, treatment, and clinical outcomes were not consistently captured. Some laboratory values in the source draft were internally inconsistent; values that could not be reconciled without the original dataset were omitted rather than inferred. These limitations restrict reproducibility and generalizability and preclude estimates of national pathogen distribution or resistance patterns.

## Conclusions

This small single-center retrospective case series describes 25 adults treated for SA at a tertiary referral hospital in Cambodia. Joint pain, fever, swelling, and knee involvement were common, and diabetes mellitus and alcohol use were frequently documented. Organisms were reported in approximately half of the patients. *B. pseudomallei *was the most frequently recorded organism, a finding that is clinically noteworthy given the established local context of melioidosis, but it should not be interpreted as evidence that *B. pseudomallei *is the predominant cause of SA nationally. The small sample, referral setting, culture-negative cases, incomplete microbiological methods, and absence of systematic prior-antibiotic and outcome data substantially limit inference. Multicenter prospective studies with standardized microbiological methods and systematic treatment and outcome collection are needed.
